# The Prospect of Improving Pancreatic Cancer Diagnostic Capabilities by Implementing Blood Biomarkers: A Study of Evaluating Properties of a Single IL-8 and in Conjunction with CA19-9, CEA, and CEACAM6

**DOI:** 10.3390/biomedicines12102344

**Published:** 2024-10-15

**Authors:** Tomas Bukys, Benediktas Kurlinkus, Audrius Sileikis, Dalius Vitkus

**Affiliations:** 1 Department of Physiology, Biochemistry, Microbiology and Laboratory Medicine, Institute of Biomedical Sciences, Faculty of Medicine, Vilnius University, LT-03101 Vilnius, Lithuania; dalius.vitkus@mf.vu.lt; 2 Clinic of Gastroenterology, Nephrourology and Surgery, Institute of Clinical Medicine, Faculty of Medicine, Vilnius University, LT-03101 Vilnius, Lithuania; benediktas.kurlinkus@mf.vu.lt (B.K.); audrius.sileikis@mf.vu.lt (A.S.)

**Keywords:** pancreatic cancer, IL-8, diagnostic biomarker, chronic pancreatitis, obstructive jaundice

## Abstract

**Background/Objectives**: This study aims to evaluate the possible clinical application of interleukin 8 (IL-8) as a single biomarker and its capabilities in combination with carbohydrate antigen (CA19-9), carcinoembryonic antigen (CEA), and carcinoembryonic antigen cell adhesion molecule 6 (CEACAM6) as diagnostic and prognostic tools for pancreatic ductal adenocarcinoma (PDAC). **Methods**: A total of 170 serum samples from patients with PDAC (n = 100), chronic pancreatitis (CP) (n = 39), and healthy individuals (n = 31) were analysed. IL-8 and CEACAM6 were measured by an enzyme-linked immunosorbent assay (ELISA). CA19-9 and CEA were determined by chemiluminescent microparticle immunoassay, and bilirubin was quantified using a diazonium salt reaction. Receiver operating characteristic curve analysis, logistic regression, and Kaplan–Meier analyses were performed to evaluate the properties of a single IL-8 and in combination with other biomarkers. **Results**: The concentrations of IL-8 were statistically significantly higher in the PDAC group compared to the CP and control groups. Heterogeneous levels of IL-8 correlated with PDAC stages (*p* = 0.007). IL-8 had good and satisfactory diagnostic efficacy in differentiating PDAC from controls (0.858; *p* < 0.001) and patients with CP (0.696; *p* < 0.001), respectively. High and low expressions of IL-8 were not significantly associated with overall survival (OS) or disease-free survival (DFS). A combination of IL-8, CEACAM6, and CA19-9 reached the highest AUC values for differentiating PDAC from the control group. The best classification score between PDAC and the control group with CP patients was obtained by merging IL-8 and CA19-9 (0.894; *p* < 0.001). **Conclusions**: These results provide compelling evidence of IL-8 as a promising diagnostic biomarker. Nonetheless, due to the high complexity of PDAC, only the conjunction of IL-8, CA19-9, and CEACAM6 integrates sufficient diagnostic capabilities.

## 1. Introduction

Pancreatic cancer is one of the most lethal gastrointestinal malignancies, which is often diagnosed in Western Europe and North America at an advanced stage in elderly patients [1,2]. Based on the recent European cancer information systems data, pancreatic cancer is ranked as the 8th most prevalent cancer in the European Union, with a hefty mortality rate of up to 94.8% of all cases [2]. The most frequently diagnosed type of pancreatic cancer is pancreatic ductal adenocarcinoma (PDAC), which has a distinctive exocrine origin, limited treatment options, and a dismal five-year survival prognosis of less than 9% [1,3]. These results reflect the late diagnosis issue. In most cases, pancreatic tumorigenesis and progression occur unnoticed for decades, while PDAC cells escape their primary location and start to circulate in the bloodstream to initiate early metastasis [4]. Moreover, PDAC is predisposed to a high-scale desmoplastic reaction, which provokes hypoxia and nutrient deprivation. Therefore, various mutations in Kirsten rat sarcoma viral oncogene homologue (KRAS), tumour suppressors (e.g., TP53, RB, and PTEN), and other canonical oncogenes (e.g., AKT, PI3K) facilitate PDAC cells to acquire biochemical flexibility by augmenting glucose, amino acids, lipids, and proteins acquisition that is linked to enhanced activity in downstream metabolic pathways. These molecular alterations can indicate potential biomarkers since normal cells flourish in a nutritious environment [1,3,5,6]. In addition, suppressed expression of human equilibrative nucleoside transporter 1 (hENT1), specific pyrimidines such as deoxycytidine, released by tumour-associated macrophages (TAMs), epithelial-mesenchymal transition phenotype, miRNA, and pancreatic stellate cells (PSCs) in the tumour stroma, through SDF-1α/CXCR4 signalling, spur multi-level chemoresistance to gemcitabine, which is widely prescribed as a first-line drug [7,8]. Currently, conventional imaging tools and gold-standard serum marker carbohydrate antigen 19-9 (CA19-9) are not utilized as screening tests for early detection of PDAC due to the costs and lack of sensitivity and specificity. Occasionally, they show false positive or false negative results that could significantly impact increasing misdiagnosis-related harm to patients [4,9]. It is thought that excessive dietary fat intake, environmental pollution, lack of physical activities, and other factors will increase the risk of developing PDAC. Hence, there is an urgent clinical need to identify non-invasive biomarkers that can be applied to diagnose PDAC and to monitor its progression or evaluate treatment effectiveness [2,3,4].

Interleukin 8 (IL-8), also known as C-X-C motif chemokine ligand 8 (CXCL8), is an 8 kDa proinflammatory molecule [10]. Expression of this chemokine can be triggered by various upstream biomolecules, such as interleukin 1 (IL-1), interleukin 6 (IL-6), tumour necrosis factor-alpha (TNF-α), interferon-γ, phytohemagglutinin, lipopolysaccharide, and others [11]. Recently, it has been presented that elevated IL-8 serum concentrations can be found in patients with PDAC [10,12]. In pancreatic cancer cells, IL-1α, through the mitogen-activated protein kinase (MAPK) cascade, stimulates the expression of IL-8 by activating ERK-1/2, p38 MAPK, activator protein-1 (AP-1), and nuclear factor kappa B (NF-κB) [13]. Further, stromal cells initiate the synthesis of IL-8, and the cancer cells secrete this chemokine in an autocrine or paracrine manner. This loop, in which IL-8 promotes ERK/MAPK activation and activator of the transcription three pathway, results in TWIST expression, thereby impacting the ability to form metastasis. In addition, expressed high levels of a hypoxia-induced factor (HIF)-1α and growth factors, for instance, epidermal growth factor, can synergistically enhance IL-8 production, thus influencing the tumour microenvironment in PDAC [6,11,13]. An in vitro study showed that IL-8 interaction with C-X-C motif chemokine receptor 1(CXCR1) complements Capan1 sphere-forming properties and contributes to cancer progression by increasing the proportion of cancer stem cells [14]. Moreover, the IL-8 tripeptide motif (i.e., Glu-Leu-Arg, ELR) and interactions with CXCR2 allow it to mimic the role of vascular endothelial growth factor (VEGF) and transactive VEGF receptor 2 to promote angiogenesis in PDAC [11,13,14]. These mechanisms substantiate the crucial role of IL-8 in the PDAC progression. Therefore, investigations have proved that dysregulation of IL-8 is associated with weight loss, lymph node or distant metastasis, rapid proliferation, and the aggressive phenotype of PDAC cells [12,13,15,16]. Litma-Zawadzka et al. demonstrated that IL-8 enables the detection of PDAC with higher specificity and sensitivity than CA19-9 or carcinoembryonic antigen (CEA) [17].

The main goals of this study were to evaluate and compare IL-8 as a single biomarker and in conjunction with CA19-9, CEA, and CEACAM6, thus determining which approach can achieve the highest diagnostic and prognostic value for patients with PDAC.

## 2. Materials and Methods

### 2.1. Study Design and Subjects

This research can be determined as a prospective study based on a cross-section model. A total of 170 blood samples were taken from patients admitted to Vilnius University Hospital Santaros Klinikos (Vilnius, Lithuania) from 2015 to 2020. Patients with histologically proven PDAC were included in this study. The control group consisted of patients who underwent treatment for haemorrhoids, inguinal hernia, etc., exhibited no signs of gastrointestinal diseases, and patients with chronic pancreatitis (CP). Applicable clinicopathological features, including tumour size, location, TNM (primary tumour, lymph node, and metastasis) stage, sex, age, and others, were documented for each patient. The Vilnius Regional Biomedical Research Ethics Committee approved this study (No.158200-13-675-214) and signed informed consent forms were collected.

### 2.2. Sample Collection, Enzyme-Linked Immunosorbent Assay (ELISA), Chemiluminescent Microparticle Immunoassay (CMIA), and Diazodium Salt Reaction

Serum samples were obtained by centrifugation for 10 min at 3000× *g* of clotted blood, collected in STT tubes (BD, Franklin Lakes, NJ, USA), and then stored at −80 °C prior to analysis. An enzyme-linked immunosorbent assay (ELISA) was used to quantitatively detect human IL-8 (Invitrogen, Vienna, Austria). Firstly, Corning Costar 9018 ELISA plates were coated with 100 µL/well of capture antibody in the coating buffer. A total of 200 µL of the ELISA/ELISPOT diluent was pipetted to the wells for 1 h at room temperature. After washing 1 time, 100 µL of the patients’ sera and prepared standards were applied for 2 h, followed by a washing step. Diluted detection antibody and Avidin-HRP were added to all wells and incubated for 1 h and 30 min, respectively. Later, 100 µL of 1X 3,3′,5,5′-tetramethylbenzidine (TMB) solution was added to each well and incubated for 15 min. Lastly, each well was filled with 100 µL of the stop solution. The plates were read with a microplate reader at 450 nm. The concentrations of the IL-8 were determined from a standard curve. The sensitivity of the IL-8 ELISA kit was 2 pg/mL. Serum concentrations of CA19-9 and CEA were determined by chemiluminescent microparticle immunoassay (ARCHITECT CA19XR (Abbott, Wiesbaden, Germany)); ARCHITECT CEA (Abbott, Sligo, Ireland) using ARCHITECT ci8200 (Abbott, Chicago, IL, USA) analyzer. The sensitivity of CA19-9 and CEA assays were >2 U/mL and >0.5 ng/mL at the 95% confidence interval (Cl), respectively. CEACAM6 concentrations were evaluated using an ELISA kit. Details of this method are described in a previously published article by Kurlinkus et al. CEACAM6 concentrations are reported in quantitative units of ng/mL [18]. Serum levels of bilirubin were detected by increased absorbance at 548 nm due to the formation of azobilirubin (ARCHITECT Total Bilirubin, Wiesbaden, Germany) using ARCHITECT ci8200 (Abbott, USA) analyzer. Bilirubin results are presented in quantitative units of µmol/L.

### 2.3. Statistical Analysis

Quantitative variables were described using means, medians, and minimum and maximum values, while categorical variables were described using absolute and percentage values. The Shapiro–Wilk test was used to define the normality of variables. The analysis showed that the distribution of IL-8, CA19-9, and CEA concentrations were not normal. Hence, the non-parametric statistical analysis was used. The Mann–Whitney U test was applied to determine the differences in biomarker concentrations between the two groups, while the Kruskal–Walli’s test was used to evaluate three or more groups. The association between categorical variables was assessed by the Chi-Squared test or Fisher’s Exact test when the expected frequency of cells was ˂5. Spearman’s rank correlation coefficient determined correlations between IL-8 and bilirubin. The diagnostic value of IL-8 was estimated by constructing a receiver operating characteristic curve (ROC). Moreover, the Youden index was applied to determine optimal cut-off values for each analyte, and other diagnostic parameters, such as sensitivity, specificity, and negative and positive predictive values, were calculated. The prognostic capabilities of IL-8 were evaluated by performing the Kaplan–Meier (log-rank test) overall survival (OS) and disease-free (DFS) analysis. Logistic regression models of CA19-9, CEA, IL-8, and CEACAM6 were designed to determine the diagnostic value differences in combined biomarkers. We adjusted CEACAM6 results based on our study cohort to include this biomarker in comparative analysis. Statistical significance was set at a *p*-value of less than 0.05. Statistical analyses were accomplished using Microsoft Excel 2311 (Microsoft Corporation, Redmond, WA, USA) and SPSS 29.0.2.0 (20) (IBM; Armonk, NY, USA).

## 3. Results

All individuals enrolled in this study were divided into three groups. The first group contained patients with PDAC. The second group consisted of patients with a diagnosis of CP. The last fraction in the cohort was denoted as the control group. All demographic parameters of patients included in the study are represented in Table 1.

**Figure 1:**
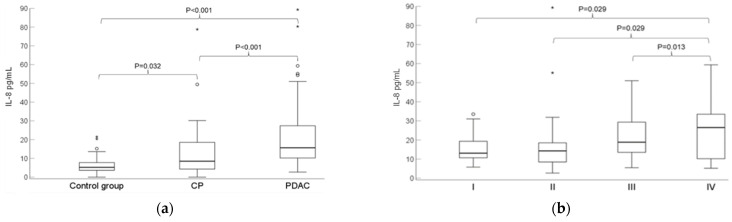
(**a**) Boxplot of IL-8 serum levels for the control group, patients with PDAC and CP. (**b**) Box plot of IL-8 serum levels among different PDAC stages (B). IL-8—Interleukin 8. PDAC—Pancreatic ductal adenocarcinoma. CP—Chronic pancreatitis. *— represent potential outliers.

An incisive comparison of the three analysed groups showed significantly higher median IL-8 concentrations in patients with PDAC (15.6 pg/mL) compared to the control (5.2 pg/mL) and CP (8.5 pg/mL) groups (Figure 1). Moreover, IL-8 concentrations in patients who have been diagnosed with stage IV PDAC were significantly higher than those of other stages (Figure 1).

The relationships between IL-8 expression and clinical data were estimated. The calculated results indicated that heterogenous IL-8 expression among PDAC stages was statistically significant (Table 2).

After determining the different patterns of IL-8 concentrations in patients with PDAC and the control group, the diagnostic efficacy of this analyte was evaluated. ROC curve analysis highlighted IL-8 as a good diagnostic biomarker that surpassed CEA but was not as precise as CA19-9 (Table 3). Logistic regression was used to construct a diagnostic model to assess whether combining IL-8, CA19-9, CEACAM6, and CEA could increase diagnostic efficiency. The regression model obtained during this analysis was as follows:

Login(P)= −5.88 + 0.236×(IL-8) + 1.06 × (CEACAM6) + 0.053 × (CA19-9) + 0.281 × (CEA)(1)

**Figure 2:**
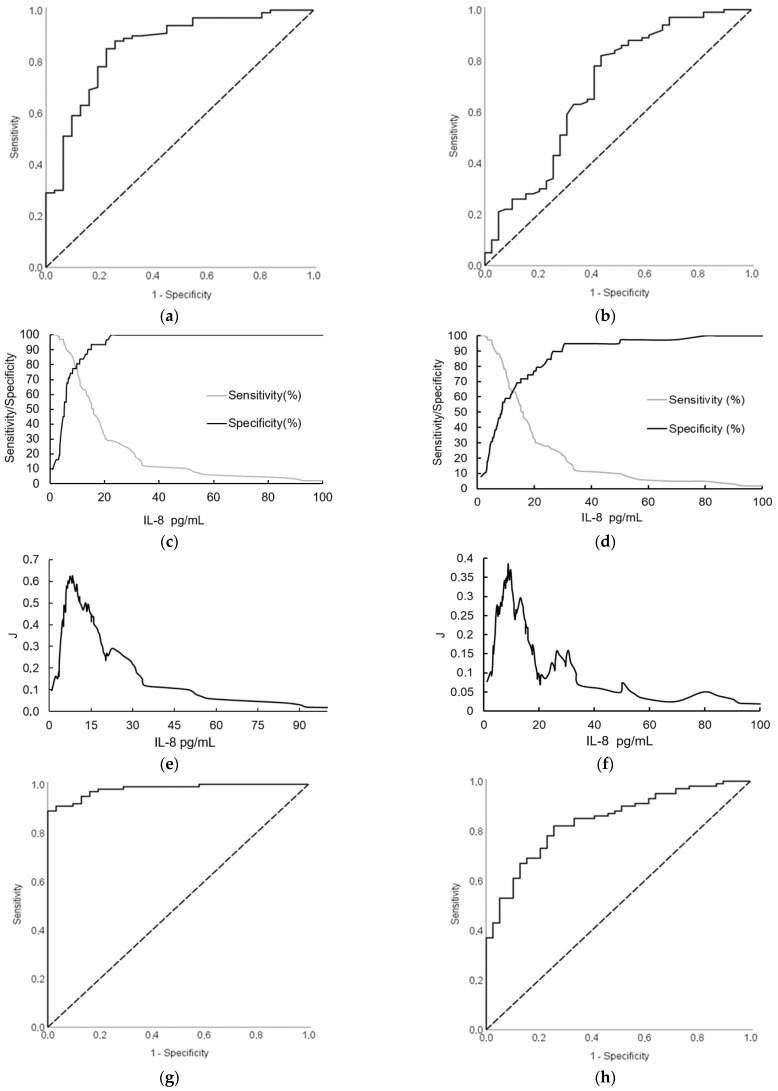
ROC curve analysis of IL-8 serum concentrations in patients with PDAC and control group or CP: (**a**) ROC curve of IL-8 (PDAC and control group); (**b**) ROC curve of IL-8 (PDAC and CP); (**c**) Specificity and sensitivity regarding serum IL-8 concentration (PDAC and control group); (**d**) Specificity and sensitivity in regarding serum IL-8 concentration (PDAC and CP); (**e**) J regarding serum IL-8 concentration (PDAC and control group); (**f**) J regarding serum IL-8 concentration (PDAC and CP); (**g**) ROC curve of all four biomarkers combined (PDAC and control group); (**h**) ROC curve of all four biomarkers combined (PDAC and CP). IL-8—Interleukin 8. J—Youden index. PDAC—Pancreatic ductal adenocarcinoma. CP—Chronic pancreatitis.

The combination of all biomarkers led to superior diagnostic efficacy for patients with PDAC (0.981; 95% CI: 0.962–0.999; *p* < 0.001) (Figure 2).

As shown in Figure 1, serum IL-8 concentrations differed among patients with PDAC and CP. Hence, we also evaluated the diagnostic efficiency of biomarkers in these two groups. The ROC analysis demonstrated that IL-8 reached moderate discrimination capabilities compared to CA19-9. However, CEA was insignificant as a biomarker (Table 3). As previously described, a diagnostic model composed of all biomarkers was designed. The equation of this model was as follows:

Login(P)= 0.323 + 0.037 × (IL-8) − 0.31 × (CEACAM6) + 0.007 × (CA19-9) + 0.055 × (CEA)(2)

The AUC value considerably increased and was 0.84 (95% CI: 0.772–0.908; *p* < 0.001) (Figure 2).

Furthermore, the sensitivity, specificity, cut-off values, and positive and negative predictive values of IL-8, CEA, CA19-9, and CEACAM6 in different groups were calculated (Table 3).

The results in Table 2 revealed that IL-8 could be a useful indicator to differentiate PDAC stages. The diagnostic efficiency between stages I-II and III-IV was evaluated to approve these findings. Comparing biomarkers separately, IL-8 had a higher AUC value of 0.671 (95% CI: 0.564–0.779; *p* = 0.003) than CA19-9 (0.528; 95% CI: 0.413–0.643; *p* = 0.626) or CEA (0.606; 95% CI: 0.495–0.717; *p* = 0.068). To increase diagnostic efficacy, an equation of all biomarkers was written as follows:

Login(P) = −1.222 + 0.006×(IL-8) + 0.153 × (CEACAM6) + 0 × (CA19-9) + 0.071 × (CEA)(3)

The AUC value increased slightly to 0.695 (95% CI: 0.592–0.798; *p* = 0.001).

To determine IL-8′s ability to predict survival in patients with PDAC, we analysed IL-8′s prognostic capabilities. Firstly, ROC analysis was performed to establish a cut-off value, which can discriminate between low and high expression of IL-8. The AUC value reached a poor classification score equal to 0.51 (95% CI: 0.381–0.632; *p* = 0.919), and the best threshold value was 26.31 pg/mL. Though these results indicated a more random predictive ability of IL-8, we investigated its prognostic value further in PDAC patients who underwent radical surgical resection. In this case, the Kaplan–Meier OS analysis was used. The established cut-off value divided 53 patients who survived more than 3 months after surgery into two groups. The Kaplan–Meier graph displayed no significant difference regarding OS and IL-8 expression prior to surgery in patients with PDAC (*p* = 0.643). Furthermore, we evaluated the time of occurrence of PDAC-free state after surgery to PDAC recurrence or death by performing a Kaplan–Meier DFS analysis. No statistically significant dependency on DFS from IL-8 was observed (*p* = 0.7). 

**Figure 3:**
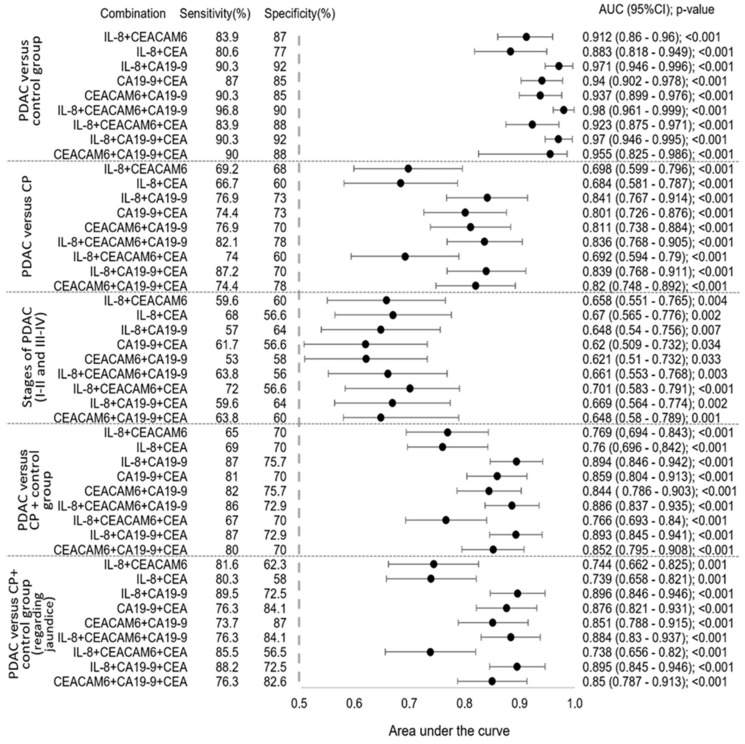
Forest plot showing the area under the biomarkers combinations efficiency curve in separate groups. In addition to the analysis, possible sensitivity and specificity were calculated for each biomarker’s combinations. IL-8—Interleukin 8; CEACAM6—Carcinoembryonic antigen cell adhesion molecule 6; CEA—Carcinoembryonic antigen; CA19-9—Carbohydrate antigen 19-9; PDAC—Pancreatic ductal adenocarcinoma; CP—Chronic pancreatitis; AUC—Area under the ROC curve; CI—Confidence interval; *p*-value—Statistical significance was set at a *p*-value of less than 0.05.

A forest plot (Figure 3) was generated to depict the straightforward comparison of two or three biomarker combinations. 

In this graphical representation, some biomarker combinations e.g., IL-8, CEACAM6, CA19-9, and IL-8 with CA19-9, achieved the highest AUC values and stood out from other combinations with the best diagnostic qualities in distinguishing patients with PDAC from the control group or patients with CP, respectively. Further, we evaluated additional parameters of these biomarkers’ combinations. IL-8 with CA19-9 compared to a single CA19-9 (0.85; 95% CI: 0.799–0.913; *p* < 0.001) or IL-8 (0.768; 95% CI: 0.693–0.843; *p* < 0.001) were more distinctly specific and sensitive approach to suspect PDAC in the control group when CP was included. In evaluating the potential to differentiate between stages of PDAC, the combination of IL-8, CEACAM6, and CEA showed the highest sensitivity and AUC value. Moreover, from clinical practice, we know that high bilirubin levels interfere with CA19-9 quantity by decreasing its specificity. To rule out or confirm if this drawback of CA19-9 applies to IL-8, a new cohort was generated by excluding patients with hyperbilirubinemia. We identified 38 CP, 76 PDAC, and 31 control group patients with ≤80 µmol/L bilirubin concentrations in serum samples. The ROC analysis evinced IL-8 as a decent stratification factor (0.743; 95% CI: 0.661–0.825; *p* < 0.001). This result was fractionally lower (3.255%) than the AUC value when all patients were included in the study. In addition, statistical analysis revealed the merits of IL-8 in conjunction with CA19-9 over a single IL-8 (Figure 3). Furthermore, Spearman’s rank correlation coefficient was used in this cohort to determine the strength of the linear relationship between IL-8 and bilirubin. No statistically significant correlation was identified (*p* = 0.462).

## 4. Discussion

Studies have shown that IL-8, known for pro-inflammatory properties, is also a paramount pro-oncogenic factor in the progression of PDAC [15,17]. Our study demonstrated that the median of IL-8 in patients with PDAC was 3 fold significantly higher compared to the control group (FIGURE 1). Our findings concur with those of other authors who detected increased levels of this chemokine in patients with PDAC diagnosis [17,19,20]. In a previously published article, IL-8 was also 3 fold significantly higher in patients with PDAC than in the control group [19]. In another study, healthy individuals who had no pyrexia within 1 week, were not pregnant, had no prescribed drugs, and had no history of chronic or acute illness were denoted as a control group. This approach could have helped to reduce the altered production of IL-8 by inflammation in the control group. As a result, IL-8 was only detected in patients with PDAC, although these findings might be inconsistent due to the lower limit of assay sensitivity, which was 5 times higher compared to the ELISA kit applied in our study [15].

In the literature, this type of pancreatic cancer is described as a poorly immunogenic tumour. However, the microenvironment, which is highly heterogeneous and characterized by one of the most abundant stromal compartments, creates a highly immunosuppressive environment. This state occurs due to the complex interaction between regulatory T cells (Tregs), TAMs, myeloid-derived suppressor cells (MDSCSs), cancer-associated fibroblast (CAF), and other cells [5,21,22]. CAFs, which can be triggered by fibroblast activation protein, alpha-smooth muscle actin, inflammation cytokines–IL-8, IL-10, and exosomes with miRNA, promote and accelerate new signalling pathway networks formation in pancreatic cancer cells [5,21,22,23]. These events imply the spark that sets off a systemic endogenous immunosuppressive state rather than local immunosuppressive status, which explicates the increased shedding of IL-8 molecules into the bloodstream when patients have PDAC [17,20]. 

However, compared to the control group, the results in our study revealed that the IL-8 concentration in the CP group was at most 1.84-fold lower than in samples from patients with PDAC. These results support the non-specific and pro-inflammatory nature of this chemokine. Thus, increased IL-8 expression can be seen in malignancies and inflammatory conditions [11]. CP can be characterized by chronic inflammation and fibrosis of the exocrine and endocrine pancreatic parenchyma, which can function as an oncogenic driver and lead to an increased risk of developing PDAC, especially in the hereditary form of the disease [24,25]. The upregulation of IL-8 expression in CP is induced by the intricate interaction of cytokines, growth factors, and crucial signalling pathways, for instance, NF-κB. These molecular mechanisms are not only predominant characteristics of chronic inflammation but also share commonalities with PDAC progression [6,11,13,14,25]. The immunohistochemistry technique facilitates the detection of IL-8 clusters in the remaining acinar cells of the exocrine parenchyma, metaplastic ductal cells, inflammatory foci, or macrophages located near enlarged pancreatic nerves [26]. In this scenario, IL-8 acts more as an inflammatory mediator, rather than a pro-oncogenic factor, that is involved in the proliferation of PDAC in harsh environments or the activation of TAMs [5,8]. These distinct roles evince the fluctuations of IL-8 concentrations in samples obtained from patients with CP and PDAC. Similar findings confirming the IL-8 diagnostic role were stated in another study. Their patients with PDAC had significantly higher serum levels of this chemokine than patients with solid pseudopapillary tumours, acute pancreatitis, or pancreatic cysts. This investigation also revealed that the AUC for diagnosing PDAC (0.71) was only 7.1% lower than our results, though they encompassed patients with pancreatic cysts and acute pancreatitis in the analysis [19].

These results confirm that IL-8 as a multifunctional component is vital for pancreatic cancer cells and has the capacity to have a higher total pool volume than in other pancreatic malignancies, colon, and gastric cancers [13,19,20]. Furthermore, based on our and previously published results, we are operating under the assumption that in some cases, IL-8 together with other diagnostic tools, for instance, computed tomography (CT) or magnetic resonance imaging (MRI) scan, abdominal or endoscopic ultrasound, lipase, and other serum parameters, might make a complementary effect. This would assist clinicians not only in diagnosing PDAC but also in predicting the possible effects of chemotherapy. In CP patients, it could help to predict clinical manifestation outcomes, for instance, abdominal pain, significantly lower scores for physical and cognitive functioning, or a higher risk of developing PDAC [24,26].

Another important finding of our study is that IL-8 concentrations were becoming higher as the stages of PDAC were increasing. An abnormal increase in IL-8 levels can already be seen in the I stage of PDAC. Tregs might likely be responsible for that. CD4 + FoxP3 + Tregs start to accumulate and secrete IL-8 and TGF-β, thus stimulating the expression of cytotoxic T-lymphocyte associated protein 4, T cell immunoreceptor with Ig, and immunoreceptor tyrosine-based inhibitory motif (ITIM) domains, etc. This leads to suppressing the activity of T-lymphocytes and natural killer cells around the tumour environment during the preinvasive stage [21,22]. These changes distinguish IL-8 as a potential biomarker that can be useful for discriminating stages of PDAC. However, the ROC analysis revealed only satisfactory capabilities for IL-8. As an alternative, emerging evidence indicates that effective early detection of PDAC may be achieved by non-coding RNA (miRNA) or closed-loop RNAs (circRNAs). A recent study analyzed 100 highly expressed miRNAs and a conjunction of those with CA19-9. The AUC value exceeded 0.9 for the miRNA profile and, combined with CA19-9, reached a superior stratification score when patients with pancreatic cancer in Stage 0–1 or Stage 0-II were compared to healthy individuals. Further investigation revealed that miRNA profile with CA19-9 enabled the screening of asymptomatic patients with pancreatic cancer in Stages 0–1 [27]. Another study suggested a circRNA panel to facilitate the early detection of PDAC. The 5-circRNA panel alone or in combination with CA19-9 exhibited high specificity and sensitivity to identify patients with early-stage PDAC or in patients characterized by the absence of CA19-9 expression [28]. These recent results imply that miRNAs or circRNA might be better approaches to tackle the problem of early detection of PDAC than IL-8. However, variability in expression, biological complexity, quantification challenges, and limited clinical data are still the main drawbacks of utilizing them in routine practice [27,28].

Kaplan–Meier OS analysis in our study showed that IL-8 is not useful as eligibility criteria to identify patients with PDAC who are more predisposed to poor survival rates. To grasp these results, we presume that, after surgery, a mass of PDAC cells that are liable to secrete IL-8 are resected; hence, the primary source of this chemokine becomes inflammatory cells [10,11,13]. This radical treatment option may be feasible to reach the most desirable outcome—remission of PDAC. Unfortunately, the vast majority of patients will develop disease recurrence and die within 5 years [1,3]. During this period, changes in IL-8 levels could be more valuable in predicting the prognosis of PDAC than in the pre-surgery period. Our findings correspond to those of Ebrahimi B. et al., who determined no correlation between IL-8 levels and survival [15]. In another study, although patients with negative IL-8 expression survived 4.8-fold longer than patients with positive IL-8 expression, no significant difference was identified [13]. Opposite results were published by Feng L. et al., who concluded that IL-8 was significant in predicting shorter overall survival time, though this study followed up a cohort of patients with TNM III and IV pancreatic cancer, and the cut-off value was selected by the median of IL-8 expression [12]. 

Diagnostic methods based on a combination of several biomarkers can ameliorate a single biomarker’s imperfections. Our previously published article focuses on estimating the probability of CEACAM6 as a biomarker for PDAC. The results suggested the prognostic value of CEACAM6 as it might predict if adjuvant chemotherapy can aggravate a patient’s condition. However, due to high CEACAM6 concentration in CP patients, this protein had low diagnostic capabilities [18]. On the other hand, IL-8 concentrations were proportionally distributed among all three groups and stages of PDAC. This indicated its capacity for higher diagnostic value. However, its main shortage might be lower specificity and sensitivity due to proinflammatory properties. These findings revealed that CEACAM6 and IL-8 as a single analyte cannot fulfil the qualities of a superb biomarker for PDAC. The necessity for a combination of biomarkers is due to the high heterogeneity observed among patients with PDAC in reference to symptoms, clinical manifestation, and predisposition to early metastasis [3,5,8,29]. Moreover, CA19-9, as a single biomarker, is not characterised as pathognomonic for PDAC. Increased levels of this analyte can be found in patients with CP, cholangitis, colorectal cancer, hepatic or pancreatic cysts, and other conditions. The association between CA19-9 and the Lewis (Le) blood group creates another formidable obstacle to its lower specificity and sensitivity [3,4,9,18,20,29].

Hence, one of the main focuses of this study was to evaluate the diagnostic efficacy of the combinations of IL-8, CA19-9, CEACAM6, and CEA. All four serum proteins let to attain the highest AUC value compared to a single biomarker. This could be a breaking point in generating a sufficient panel of analytes that incorporates prognostic and diagnostic properties for PDAC patients’ management. However, meeting the crucial requirements for a beneficial biomarker that can be implemented in clinical practice would be difficult. Testing all four biomarkers would be arduous for the laboratory due to different analysis methodologies. Screening a healthy or a high-risk population for the foreseeable presence of PDAC would not be cost-effective, and interpreting these results would be a grossly excessive task for regular clinicians [19,20,29]. 

Alternatively, from our data generated during this research, a panel of two or three biomarkers can render identical or even higher diagnostic and prognostic efficiency. Our findings correspond to those of Litman-Zawadzka et al., who analysed the diagnostic sensitivity of IL-8 and classic tumour biomarker combinations. Compared to our results, IL-8 and CA19-9 achieved even better diagnostic efficacy, although the cohort was 1.72-fold lower than ours, and the diagnostic sensitivity was determined based on the percentage of elevated concentration of IL-8 [17]. Another group described that IL-8 merged with other biomarkers was imperative to enable the most accurate discrimination of PDAC. Their study identified a panel of IL-8, interferon gamma-induced protein 10 (IP-10), IL-1b, and platelet-derived growth factor (PDGF) as having a more significant impact on classifying patients with PDAC and benign disease in the presence of obstructive jaundice compared to CA19-9 [20]. However, we assumed that a higher cut-off value of bilirubin, for instance, ≥80 µmol/L, would be more appropriate to reflect the presence of obstructive jaundice, and patients with different benign diseases of the pancreas, e.g., choledocholithiasis, CP, choledochal cyst, post-cholecystectomy strictures, etc., should be enrolled [20,30]. In our study, the interference effect from bilirubin was evaluated by excluding patients with hyperbilirubinemia. This approach allowed us to analyse IL-8 from a different perspective. 

Specifically, CT as a first-line imaging technique serves to accentuate the distinctive nature of PDAC [1,3,31]. These structural and functional abnormalities caused by metabolic alterations in PDAC cells limit more extensive use of CA19-9 [5,9,21]. Abnormally excessive cell proliferation is accompanied by altered expression levels of CA19-9. Further, mechanical obstruction of the biliary duct and secondary inflammations in PDAC upregulate secretion of CA19-9 by normal biliary or pancreatic ductal epithelium, resulting in increased shedding of this protein into the bloodstream. Therefore, various sources of this biomarker decrease its diagnostic capacity for malignant obstructive jaundice and benign obstructive jaundice [9,30,31,32]. In contrast, our findings indicated that IL-8 is a more independent differential factor regarding hyperbilirubinemia in PDAC. These results, in conjunction with imaging techniques, highlight the more versatile use of IL-8 in the clinical workflow when other pancreatic lesions, for instance, high-grade neuroendocrine tumours, metastases, etc., mimic PDAC [19,30,33].

To make our and other groups’ presumptions of biomarker combinations possible in clinical practice, we consider a more standardised method to secure reproducibility, repeatability, exceptional selectivity, and sensitivity for quantifying IL-8 and CEACAM6. The immunoassay used in the current and previous studies had high sensitivity and specificity properties. However, this method, in some cases, tends to provide false positive or negative results, which are not a desirable characteristic [34]. To solve these flaws, we hypothesise that analytical chemistry techniques—liquid chromatography with tandem mass spectrometry (LC-MS/MS)—could increase the detection limit and add a dimensionless quantity—m/z (mass/charge). CEACAM6 contains an N-terminal Ig variable-region-like (IgV) domain, which renders them to form homodimers and heterodimers with CEACAM1 and CEACAM5, which are also expressed in PDAC. Therefore, not only anatomical barriers that surround host tissues and endothelial cells but also a complexation of non- and identical monomers could contribute to lower levels of the pool of free CEACAM6 in the bloodstream. Some studies imply that during glycosylation of CEACAMs, a folded yet in-active CEACAM molecule forms to inhibit interactions with other molecules. However, what forms of CEACAM6 dominate in a PDAC patient’s blood or tissue samples are unknown [18,35,36]. 

Similar problems on a molecular basis that can affect the sensitivity and specificity of IL-8 can be related to this analyte biochemical structure. As described in the introduction section, depending on the stimulus, e.g., cytokine or cellular stress, different sparks from various intracellular signalling pathways are responsible for the transcriptional regulation of IL-8. Transcripts of the IL-8 gene provide a template to encode the precursor protein of 99 amino acids. After synthesis, two biologically relevant isoforms of IL-8 are formed. The first variant comprises 72 amino acids [ser-IL-8]72, mainly secreted by monocytes, neutrophils, lymphocytes, and macrophages. The other form has five extra amino acids [ala-IL-8]77 and is expressed in non-immune cells. A longer peptide chain enriches the IL-8 capabilities by acquiring distinct pro-apoptotic and chemotactic activity for malignant cells. Studies have revealed that during human development, the composition of these two isoforms differs in contrast to the required specific functionality [10,11,13,26,37]. However, to our knowledge, no study has been conducted to evaluate the predominant form or possible differential expression of IL-8 isoforms during the development and progression of PDAC. We assume that, in this scenario, the calculated ratio of isoforms could facilitate new insight into IL-8 as a universal biomarker to diagnose or predict the course of PDAC and add a fundamental value to a combination with CEACAM6 and CA19-9. However, some may argue with our suggested approach to using an LC-MS/MS system to measure IL-8 and CEACAM6 due to its complexity and price compared to conventional methods used in clinical laboratories. To rule out these concerns, we have estimated the cost of care and treatment for PDAC patients. In 2022, 605 new cases of pancreatic cancer were identified in Lithuania [2]. A recent study has found that European countries’ average direct and indirect costs can reach up to EUR 194614 per patient. Based on these numbers, about EUR 117.74 million had to be distributed to all Lithuanian PDAC patients [2,38]. This is a significant expenditure on a healthcare budget, which could be reduced by diagnosing the disease at earlier stages and improving treatment outcomes [3,10,17].

Certain limitations of this study design may have affected the accuracy of the results. First, the number of individuals varied between all three groups. The control group and the number of patients with CP were 3.22-fold and 2.57-fold lower, respectively, than those who were diagnosed with PDAC. In addition, data on gender, BMI, age, etc., were heterogeneous. For instance, the difference between the control and PDAC groups’ median age was more than 10 years. These discrepancies may have affected the results. Secondly, this research was not conducted as a continuous observation. This does not let us evaluate the effect of time. Considering this, the dynamic data on patients’ clinical status and IL-8 concentrations at various time points should be collected to assess the likelihood of predicting response to a specific treatment, tumour progression, remission, or recurrence. Moreover, our study did not consider the stability of IL-8 in serum samples. To our knowledge, no available data exists on this protein’s immunoreactivity changes when samples are frozen at −80 °C. ELISA IL-8 kit manufacturer does not specify the exact shelf-life or freeze-thaw cycle count and how these factors can affect results. In our case, the quantitative analysis of IL-8 was performed during the COVID-19 pandemic, when the bulk of manufacturers faced supply chain disruptions, which caused reagent kit delivery delays to our laboratory. Consequently, all analyses were not conducted at the same time as planned, and samples were repeatedly frozen and stored for more than one year. In conjunction, we hypothesise that repeated freeze-thaw cycles can cause ice crystal formation in samples, thereby impacting more rapid IL-8 degradation. However, further investigations in this field need to be performed to confirm or deny our presumption. 

## 5. Conclusions

In conclusion, our study suggests that proinflammatory interleukin 8 can be deemed as a promising biomarker for diagnosing PDAC. Its diagnostic value increases even more when used with conventional biomarkers, as IL-8 and CA19-9 achieved the highest AUC value. Further, IL-8 has properties that evaluate the risk of PDAC in patients with obstructive jaundice, and this characteristic improves when a panel comprised of IL-8 and CA19-9 is utilised. However, IL-8 alone or with other biomarkers could not make a complementary contribution to predicting the stage of PDAC without conventional imaging tools. 

## Figures and Tables

**Table 1:** Demographic parameters of patients included in the study.

Variable	PDAC ^1^	Chronic Pancreatitis	Control Group
Total number	100	39	31
Male	51	33	14
Female	49	6	17
Average age (years)	66	49	55
BMI ^2^ (kg/m^2^)	25.97	22.56	28.3

^1^ PDAC—Pancreatic ductal adenocarcinoma; ^2^ BMI—body mass index.

**Table 2:** Association between IL-8 levels and clinicopathological features in patients with PDAC.

Variable		Number (N)	Median	(min-max)	*p*-Value
Age (years)	<60	26	16.46	2.67–89.33	0.842
	>60	74	15.17	3.5–94.3	
Sex	Female	49	12.67	3.5–80.33	0.294
	Male	51	16.09	2.67–94.3	
Tumor size	<4 cm	61	15.17	3.5–94.3	0.350
	>4 cm	39	17.07	2.67–80.33	
Location	Head	76	16.09	3.5–94.3	0.43
	Body	13	14.33	3.5–33.5	
	Tail	11	15.17	2.67–35.18	
TNM Stage	I	12	13.08	5.79–33.5	0.007
	II II	41	14.27	2.67–89.33	
	III	25	18.82	5.48–94.3	
	IV	22	26.53	5.2–59.3	
T ^1^	1c	5	15.2	11–54.33	0.375
	2	48	16	3.5–94.3	
	3	24	12.67	2.67–55.17	
	4	23	17.45	5.17–91.83	
N ^2^	0	27	14.3	2.67–55.17	0.099
	1	54	14.33	3.5–89.33	
	2	19	19.42	8.5–94.3	
M ^3^	0	78	15.17	2.67–94.3	0.127
	1	22	26.53	5.17–59.33	
G ^4^	2	58	16	2.67–94.3	0.314
	3	48	15.17	5.17–91.83	
R ^5^	0	35	13.08	2.67–89.33	0.189
	1	23	18.5	3.5–94.3	
BMI ^6^	<25	45	15.6	2.67–94.3	0.648
	25–30	38	15.17	5.48–91.83	
	>30	16	15.21	5.17–32.45	

^1^ primary tumor; ^2^ regional lymph nodes; ^3^ distant metastasis; ^4^ differentiation; ^5^ resection status; ^6^ BMI—body mass index.

**Table 3:** Parameters of IL-8, CA19-9, CEACAM6, and CEA in different groups.

Parameters	PDAC ^1^ Patients and Control Group				PDAC and CP ^2^ Patients			
	IL-8 ^3^	CA19-9 ^4^	CEA ^5^	CEACAM6 ^6^	IL-8	CA19-9	CEA	CEACAM6
AUC ^7^ (95%CI ^8^)	0.858 (0.781–0.935)	0.915 (0.867–0.963)	0.713 (0.619–0.818)	0.783 (0.689–0.877)	0.696 (0.59–0.801)	0.81 (0.734–0.866)	0.482 (0.381–0.583)	0.365 (0.262–0.468)
*p*-value	<0.001	<0.001	<0.001	<0.001	<0.001	<0.001	0.739	0.01
Cut-off value	7.48 pg/mL	9.13 U/mL	1.55 ng/mL	1.21 ng/mL	8.98 pg/mL	12.9 U/mL	1.6 ng/mL	2.22 ng/mL
Sensitivity (%)	88	88	67	88	81	80	44	50
Specificity (%)	74.2	80.6	61.3	61.3	56.4	61.5	56.4	17.9
Positive prognostic value (%)	91.7	93.6	90.91	88.5	82.7	84	78.12	75.76
Negative prognostic value (%)	65.7	67.6	53.45	72.09	53.7	54.5	51.32	43.82

^1^ PDAC—Pancreatic ductal adenocarcinoma; ^2^ CP—Chronic pancreatitis; ^3^ IL-8—Interleukin 8; ^4^ CEA—carcinoembryonic antigen; ^5^ CA19-9—Carbohydrate antigen 19-9; ^6^ CEACAM6—Carcinoembryonic antigen cell adhesion molecule; ^7^ AUC—The area under the ROC curve; ^8^ CI—Confidence interval.

## Data Availability

Patients’ clinical and demographical data presented in this study could be available on request from the corresponding author. The data are not publicly available due to privacy issues.
